# *In Vitro* Effects of Live and Heat-Inactivated *Bifidobacterium animalis* Subsp. *Lactis*, BB-12 and *Lactobacillus rhamnosus* GG on Caco-2 Cells

**DOI:** 10.3390/nu12061719

**Published:** 2020-06-08

**Authors:** Vivian M. Castro-Herrera, Christine Rasmussen, Anja Wellejus, Elizabeth A. Miles, Philip C. Calder

**Affiliations:** 1 School of Human Development and Health, Faculty of Medicine, University of Southampton, Southampton SO16 6YD, UK; eam@soton.ac.uk (E.A.M.); P.C.Calder@soton.ac.uk (P.C.C.); 2 Chr. Hansen A/S, 2970 Hoersholm, Denmark; DKCHRA@chr-hansen.com (C.R.); DKAWE@chr-hansen.com (A.W.); 3 NIHR Southampton Biomedical Research Centre, University Hospital Southampton NHS Foundation Trust and University of Southampton, Southampton SO16 6YD, UK

**Keywords:** probiotic, gut epithelium, inflammation, *B. animalis* subsp. *lactis*, BB-12, *L. rhamnosus GG*, heat-inactivation

## Abstract

Probiotic–host interaction can be cell-to-cell or through metabolite production. Dead (inactive) organisms could interact with the host, leading to local effects and possible health benefits. This research examined the effects of live and heat-inactivated *Bifidobacterium animalis* subsp. *lactis*, BB-12 (BB-12) and *Lactobacillus rhamnosus GG* (LGG) on cultured Caco-2 cells focusing on epithelial integrity and production of inflammatory mediators. Live organisms increased transepithelial electrical resistance (TEER), a barrier-integrity marker, with LGG having a greater effect than BB-12. When mildly heat-treated, both organisms had a more modest effect on TEER than when alive. When they were heat-inactivated, both organisms had only a limited effect on TEER. Neither live nor heat-inactivated organisms affected production of six inflammatory mediators produced by Caco-2 cells compared to control conditions. Pre-treatment with heat-inactivated LGG or BB-12 did not alter the decline in TEER caused by exposure to an inflammatory cocktail of cytokines. However, pre-treatment of Caco-2 cells with heat-inactivated organisms alone or their combination decreased the production of interleukin (IL)-6, IL-18, and vascular endothelial growth factor. To conclude, while the live organisms improve the epithelial barrier using this model, neither live nor heat-inactivated organisms directly elicit an inflammatory response by the epithelium. Pre-treatment with heat-inactivated BB-12 or LGG can reduce some components of the response induced by an inflammatory stimulus.

## 1. Introduction

The World Health Organization defines probiotics as “live microorganisms which when administered in adequate amounts confer a health benefit on the host” [1]. This definition assumes that probiotics need to be alive to interact with the host in order to exert benefits on health. Certainly, interactions of live probiotics with the host’s gastrointestinal epithelium and immune system are key to many of the ascribed clinical benefits, such as remission of active ulcerative colitis [2] and control of pathogenic intestinal bacterial overgrowth [2,3]. The probiotic–host interaction can be a direct cell-to-cell physical one (e.g., through pili interactions with host cells) [4,5] or can be as a result of metabolites or other products released by the probiotic organisms [6,7]. However, live organisms may also have adverse effects, as reported in case studies of microbial appearance in liver biopsies in older individuals [8] and systemic infections with probiotic organisms [9]. Dead or inactive organisms could also interact with the host and be functional, thus conferring health benefits, and would not carry the risk of infection. Both live and heat-killed organisms (a mixture of probiotics consisting of *Lactobacillus plantarum*, *L. bulgaricus*, *L. casei*, *L. acidophilus*, *Bifidobacterium breve*, *B. longum* subsp. *longum*, *B. longum* subsp. *infantis* and *Streptococcus salivarus* subsp. *thermophilus*) had significant anti-inflammatory effects through the reduction of interleukin (IL)-6 in an experimental model of colitis in rats [10]. In vitro studies showed that the anti-inflammatory effects of live and heat-killed *B. breve* were comparable in peripheral blood mononuclear cells isolated from patients with ulcerative colitis [11]. One reason that heat-killed or inactivated organisms may retain activity is that they keep the integrity of the cell wall components involved in interactions with the host [12]. In humans, particularly in vulnerable subgroups such as the frail elderly or critically ill, heat-inactivated probiotics may be a safer alternative to live organisms, as they can elicit local benefits [13]. Theoretically, local effects of heat-inactivated probiotics can translate into systemic benefits, but this requires further exploration. Here, we examined the effects of live and heat-inactivated *B. animalis* subsp. *lactis*, BB-12 and *L. rhamnosus* GG on cultured Caco-2 cells (a human colonic enterocyte cell line) with a focus on barrier integrity and production of inflammatory mediators. Both the direct effects of the organisms and the effects of pretreatment with the organisms on the subsequent response to inflammatory stimulation were examined. We chose to investigate the effects of *B. animalis* subsp. *lactis*, BB-12 and *L. rhamnosus* GG because they are widely used in the food industry and because the combination of these two organisms is currently being tested in a large clinical trial [14]. A previous study noted that the ability of probiotics to interact with Caco-2 cells is strain dependent, but that lactobacilli and bifidobacteria could directly elicit a low level inflammatory response in Caco-2 cells [15]. We used Caco-2 cells because they are considered suitable to assess the interaction between microorganisms and the gut epithelium [16].

## 2. Materials and Methods

Two experiments were carried out to study the effect of viable and heat-inactivated *L. rhamnosus* GG and *B. animalis subsp. lactis,* BB-12 on transepithelial electrical resistance (TEER) and secretion of inflammatory mediators under control and inflammatory conditions.

### 2.1. Preparation of Heat-Inactivated and Live L. rhamnosus GG and B. animalis subsp. Lactis, BB-12

*L. rhamnosus GG* and *B. animalis* subsp. *lactis,* BB-12 (Chr. Hansen A/S, Hoersholm, Denmark) were inactivated by exposure to heat; different durations of exposure were used in order to identify the best condition to inhibit colony formation indicative of inactivation. *L. rhamnosus* GG and *B. animalis* subsp. *lactis*, BB-12 were inoculated from frozen stock and cultured overnight at 37 °C in De Man, Rogosa and Sharpe (MRS) broth, pH 6.5 (Difco™), with 0.05% cysteine hydrochloride monohydrate (CyHCl) under anaerobic conditions with AnaeroGen pads (Oxoid). Ten-fold dilution series were prepared from the overnight cultures and incubated overnight at 37 °C under anaerobic conditions. For each strain, late exponential/early stationary phases were selected based on measures of optical density at 600 nm (OD600). Bacterial cultures were washed twice in 37 °C preheated Hanks’ balanced salt solution (HBSS; Gibco™) and once in antibiotic free cell culture medium (Minimum Essential Medium (MEM; Gibco™) including 20% heat-inactivated fetal bovine serum (FBS; Gibco™) and 1% MEM non-essential amino acids (Biowest, Nuaille, France). Samples were centrifuged at 3500 × *g* for 5 min. The medium was removed, and the cultures were resuspended in 5 mL antibiotic free cell culture medium. OD600 was adjusted to 3.8, and each cell suspension was divided into multiple aliquots of 1.5 mL in Eppendorf tubes. Viable *B. animalis subsp. lactis,* BB-12 and *L. rhamnosus* GG were used directly in experiments. Heat inactivation of *B. animalis* subsp. *lactis,* BB-12 and *L. rhamnosus* GG was tested at 62.3 °C for 0, 2, 4, 6, and 8 min, and heat inactivation of *L. rhamnosus* GG was tested at 70 °C for 1, 3, and 5 min by the use of a temperature-controlled waterbath. The degree of heat inactivation was assessed by subsequent growth on MRS agar (Difco™) and counting the colony forming units. In short, 1 mL of the resuspended bacterial cells was diluted in 9 mL of MRD Maximum Recovery Diluent (Dilucup^®^; LabRobot, Stenungsund, Sweden), and a 10-fold dilution series was prepared using Dilucups and the Dilushaker system. For each dilution, duplicate MRS agar plates were prepared by adding 1 mL of sample from the Dilucup and deep seeding in melted MRS agar including 0.05% CyHCl. Plates were incubated anaerobically with AnaeroGen pads for 2 days at 37 °C, and colonies were counted. Only plates with colony counts between 20 and 300 colonies were used for calculating CFU. 

### 2.2. Experiment 1

In this experiment, *L. rhamnosus* GG and *B. animalis* subsp. *lactis,* BB-12 were tested as viable and heat-inactivated cells; TEER was assessed, and the concentrations of inflammatory mediators in the medium from the apical and basolateral sides of Caco-2 cell cultures were measured.

#### 2.2.1. Culturing of Caco-2 Cells

The human intestinal epithelial Caco-2 cell line (DSMZ ACC 169, Leibniz-Institut DSMZ-Deutsche Sammlung von Mikroorganismen und Zellkulturen GmbH, Braunschweig, Germany) was cultured in MEM (Gibco™) supplemented with 20% heat-inactivated FBS (Gibco™), 1% MEM non-essential amino acids (Biowest, Nuaille, France), and 1% Pen-Strep-Amp B (Biological Industries, Cromwell, CT, USA) at 5% CO_2_ and 37 °C. When the cells were approximately 50% confluent, the medium was removed, and the cells were washed twice in Hanks’ balanced salt solution (HBSS; Gibco™). The cells were trypsinized by adding 2 mL of TrypLE Express Enzyme (Gibco™) and left for 4 min in the CO_2_ incubator at 37 °C. Approximately 10 mL of medium was added to the trypsinized cells; they were counted, and a concentration of 1 × 10^5^ cells/mL in supplemented MEM was prepared. A volume of 500 µL of cell suspension was used to seed each apical compartment of Transwell^®^-Clear Inserts, Polyester Membranes (12 mm, 0.4 µM, Corning^®^, Wiesbaden, Germany), and then 1.5 mL of supplemented MEM was added to the basolateral compartment. Cells (Passage 3) were cultured on the inserts for 21 days with change of medium twice a week. After 21 days, the transwells were moved to the CellZscope2 (NanoAnalytics, Munster, Germany). The CellZscope is a computer-controlled multi-well module with dynamic measuring of the TEER without removing the cells from the incubator; TEER is measured by applying weak alternating current voltage, unharmful to the cells, over the Caco-2 layer. The medium was changed to antibiotic-free medium adding 1.65 mL and 0.76 mL of antibiotic-free medium in the basolateral and the apical compartments, respectively. The CellZscope2 was placed overnight in a CO_2_ incubator (5%) at 37 °C, and TEER (Ω × cm^2^) was measured every hour using automated data collection. This overnight measurement of TEER before the experimental start allowed for determination of baseline TEER in each well and served as a quality control of a stable electrical resistance.

#### 2.2.2. Stimulation of Caco-2 Cells with Live and Heat-Inactivated *B. animalis subsp. lactis*, BB-12 and *L. rhamnosus* GG

*B. animalis* subsp. *lactis,* BB-12 heat-inactivated for 4 and 6 min at 62.3 °C and *L. rhamnosus* GG heat-inactivated for 1, 3, and 5 min at 70 °C as well as live bacteria were selected for testing in the CellZscope2. In order to stimulate the Caco-2 cells with bacteria, CellZscope2 measurements were paused, the CellZscope2 was removed from the CO_2_ incubator, and 100 µL of apical medium was removed from each transwell. A 100 µL of bacteria suspension (final OD600 nm of 0.5) or medium control (antibiotic-free MEM) was added to the apical side of the relevant wells (each in triplicate). The CellZscope2 was transferred back to the CO_2_ incubator, and the TEER measurements were resumed and continued overnight. Changes in TEER during bacterial stimulation were calculated relative to the latest value recorded immediately prior to the stimulation (baseline measurement, set to 100%). Area under curve (AUC) was calculated for each condition. Once TEER measurements were completed (after 24 h) apical and basolateral media were collected to analyze the concentrations of an inflammatory panel consisting of six inflammatory mediators: IL-6, IL-18, IL-8, interferon gamma-induced protein 10 (IP-10), vascular endothelial growth factor (VEGF), and intercellular adhesion molecule-1 (ICAM-1) (Magnetic multiplex immunoassay-Bio-Rad Luminex Analyzer).

### 2.3. Experiment 2

In this experiment, heat-inactivated *L. rhamnosus* GG and *B. animalis* subsp. *lactis,* BB-12 were tested; TEER was assessed, and the concentrations of inflammatory mediators in the medium from the apical and the basolateral sides of Caco-2 cell cultures were measured following stimulation with an inflammatory cocktail. 

#### 2.3.1. Culturing of Caco-2 Cells

Caco-2 cells were obtained from the European Collection of Authenticated Cell Cultures (a Culture Collection of Public Health England, CACO-2 ECACC 86010202, Human colon adenocarcinoma) and were grown in Dulbecco’s modified Eagle’s medium supplemented with 10% fetal bovine serum, 1% nonessential amino acids, 2% L-glutamine, and 1% penicillin-streptomycin (Sigma Aldrich, Gillingham, UK), at 37 °C in an atmosphere of 5% CO_2_ and 95% air, using polystyrene cell culture flasks (Sigma-Aldrich) according to methods described elsewhere [17,18]. Confluent cell cultures (Passage 46 to 48) were used after 19 days. Cells were detached from flasks using 2.5% trypsin in Hank’s balanced salt solution containing 0.2 g ethylenediaminetetraacetic acid per liter (all from Sigma-Aldrich). Subsequently, trypsin was neutralized with pre-warmed supplemented medium, and the cells were transferred to 12 insert transwell plates (12 mm with 0.4 µm clear pore size). Supplemented medium was added to both the apical (500 µL) and the basolateral sides (1500 µL) of the cultures, which were then placed at 37 °C in an atmosphere of 5% CO_2_ and 95% air. Medium on both sides of the transwell was replaced every second day.

#### 2.3.2. Effect of Pre-Treatment of Caco-2 Cells with Heat-Inactivated *L. rhamnosus* GG and *B. animalis* subsp. *Lactis*, BB-12 on Caco-2 Cell Response to an Inflammatory Cocktail

Caco-2 cell monolayers (7 × 10^4^ cells/well) were incubated in transwell plates with heat-inactivated *L. rhamnosus* GG (heat-inactivated for 3 min at 70 °C) or *B. animalis* subsp. *lactis,* BB-12 (heat-inactivated for 6 min at 62.3 °C) or the combination of *L. rhamnosus GG + B. animalis* subsp. *lactis,* BB-12 (same heat inactivation conditions) for 24 h; the bacteria were added on the apical side of the wells at a multiplicity of infection of 10:1. Then, an inflammatory cocktail of tumor necrosis factor (TNF)-α (1 ng/mL), interferon (IFN)-γ (10 ng/mL), and IL-1β (1 ng/mL) was added as a pre-warmed mixture in supplemented medium on the basolateral side of the cells. The cultures were incubated for 24 h; control cultures were not pretreated with heat-inactivated probiotics. TEER was measured using “chopstick” electrodes (see below), and apical and basolateral media were collected to analyze the concentrations of an inflammatory panel consisting of six mediators: IL-6, IL-18, IL-8, IP-10, VEGF, and ICAM-1 (Magnetic multiplex immunoassay-Bio-Rad Luminex Analyzer).

#### 2.3.3. TEER Measurement

In this experiment, TEER was measured using an epithelial voltohmeter; this instrument uses a pair of electrodes (“chopsticks”), which are placed in the transwell (Milicell ERS-2 Voltohmmeter; World Precision Instruments, Hitchen, UK). One electrode is in contact with the basolateral culture medium, and the shorter electrode is placed on top of the actual membrane where cells are seeded. Cells are never in contact with the electrodes. The calibrations of the instruments and performance of the technique were carried out according to the manufacturer’s instructions.

### 2.4. Measurement of Inflammatory Mediators

Cell supernatants were kept at −80 °C until processing. When ready to use, supernatants were defrosted, vortexed, and centrifuged for 30 s to remove any particulate matter. Cell supernatants were diluted 1:2 in buffer immediately before assay. Microparticles were resuspended in buffer and read using a pre-calibrated Bio-plex Luminex Analyzer (Bio-Plex 200, Bio-Rad, Watford, UK). The inflammatory panel assessed consisted of six inflammatory mediators whose sensitivity values (pg/mL) were: IL-6 (1.7), IL-18 (1.93), IL-8 (1.8), IP-10 (1.18), VEGF (2.1), and ICAM-1 (87.9). Measurements were carried according to the manufacturer’s instructions (Magnetic multiplex immunoassay; R&D Systems, Abingdon, UK).

### 2.5. Statistics

Data were analyzed by one-way analysis of variance by ranks (ANOVA) performed using Prism (Version 8.0, San Diego, CA, USA). Dunnet’s test was used to make pairwise post-hoc comparisons. In all cases, a value for *p* < 0.05 was considered to indicate statistical significance.

## 3. Results

### 3.1. Heat-Inactivation of L. rhamnosus GG and B. animalis subsp. Lactis, BB-12

Table 1 shows the degree of inactivation of *L. rhamnosus* GG and *B. animalis* subsp. *lactis*, BB-12 that was achieved at the selected temperatures and timepoints. Treatment at 62.3 °C for 4 min knocked down *B. animalis* subsp. *lactis*, BB-12 by more than three logs, whereas 6 min exposure at this temperature resulted in no live *B. animalis* subsp. *lactis*, BB-12 remaining. In the case of *L. rhamnosus* GG, 62.3 °C for 6 min reduced live numbers by 85%, while after 8 min, live numbers were reduced by 97%. Thus, *B. animalis* subsp. *lactis*, BB-12 is more heat sensitive than *L. rhamnosus* GG. Incubating *L. rhamnosus* GG at 70 °C for 1 min reduced live numbers by 38%, while incubation for 3 min resulted in no live *L. rhamnosus* GG remaining.

### 3.2. Effect of Live and Heat-Inactivated L. rhamnosus GG and B. animalis subsp. lactis, BB-12 on TEER in Caco-2 Cell Monolayers

**Figure 1:**
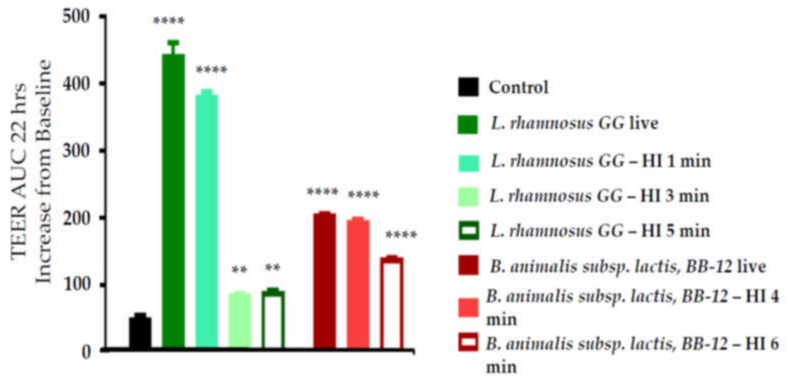
Transepithelial electrical resistance (TEER) area under curve (AUC) change after treatment of Caco-2 cells on the apical side with live or heat-inactivated (HI) *L. rhamnosus* GG or *B. animalis* subsp. *lactis,* BB-12 for 22 h. Data are mean + SD (*n* = 3). One-way ANOVA *p* value < 0.0001. ** *p* < 0.01; **** *p* < 0.0001 vs. control.

Figure 1 shows the TEER area under the curve (AUC) from 0–22 h. *L. rhamnosus* GG induced a greater TEER effect than *B. animalis* subsp. *lactis,* BB-12. Heat inactivation of both organisms reduced the ability to increase TEER compared to the live strains, but TEER was still significantly improved compared to control conditions. TEER remained higher with heat-inactivated *B. animalis* subsp. *lactis,* BB-12 than with heat-inactivated *L. rhamnosus* GG.

### 3.3. Effect of Live and Heat-Inactivated L. rhamnosus GG and B. animalis subsp. Lactis, BB-12 on Inflammatory Mediator Production in Caco-2 Cell Monolayers

**Figure 2:**
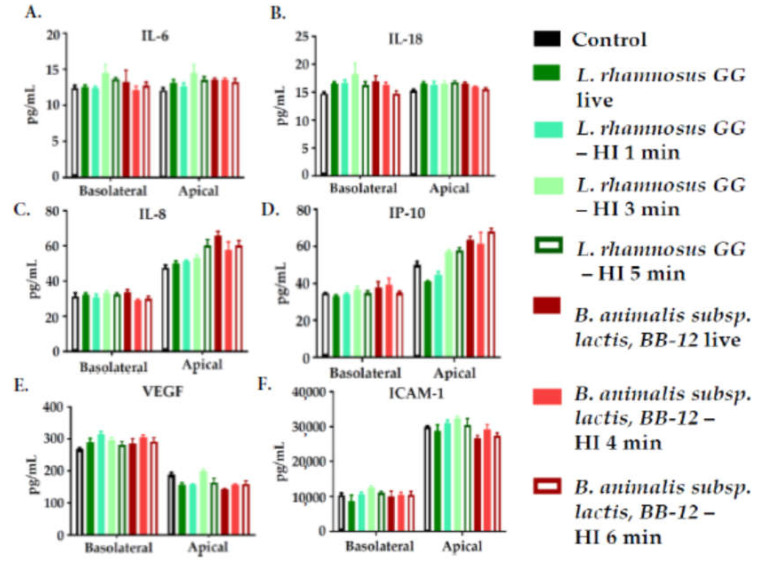
Concentrations (pg/mL) of IL-6 (**A**), IL-18 (**B**), IL-8 (**C**), IP-10 (**D**), VEGF (**E**), and sICAM-1 (**F**) in the apical and the basolateral medium of Caco-2 cells treated on the apical side with live or heat-inactivated (HI) *L. rhamnosus* GG or *B. animalis* subsp. *lactis,* BB-12 for 22 h. Data are mean + SEM (*n* = 4). Data for apical and basolateral sides were analyzed separately by one-way ANOVA (all *p* > 0.05).

Six inflammatory mediators (IL-6, IL-8, IL-18, IP-10, VEGF, and sICAM-1) were measured in culture medium collected from the apical and the basolateral sides of Caco-2 monolayers treated with live or heat-inactivated *L. rhamnosus* GG or *B. animalis* subsp. *lactis*, BB-12 for 22 h (Figure 2). IL-6 and IL-18 concentrations were similar on both apical and basolateral sides. IL-8, IP-10, and sICAM-1 concentrations were higher on the apical than the basolateral side. VEGF concentrations were higher on the basolateral than the apical side. Neither live nor heat-inactivated *L. rhamnosus* GG or *B. animalis* subsp. *lactis*, BB-12 altered the concentration of these mediators (Figure 2).

### 3.4. Effect of Pre-Exposure to Heat-Inactivated L. rhamnosus GG and B. animalis subsp. lactis, BB-12 on the Caco-2 Cell TEER Response to an Inflammatory Cocktail

**Figure 3:**
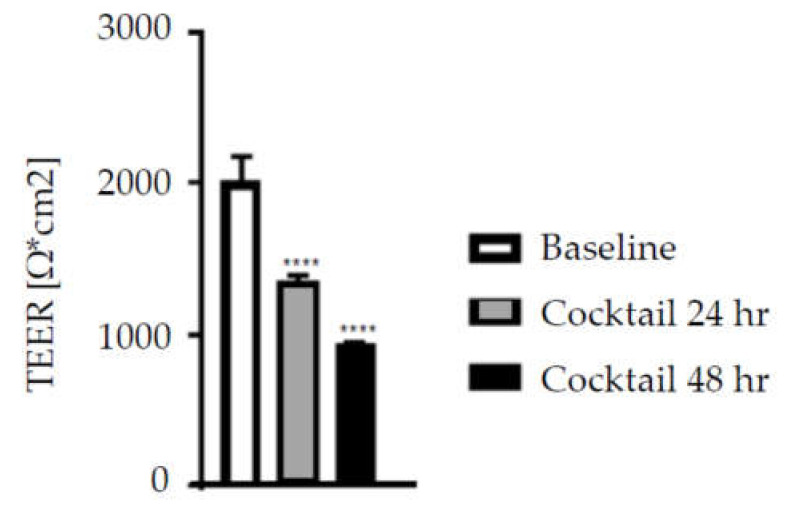
TEER measurements of Caco-2 cell cultures exposed to a cocktail of inflammatory cytokines on the basolateral side for 24 or 48 h. Data are mean + SEM (*n* = 9). **** *p* < 0.0001 vs. baseline.

**Figure 4:**
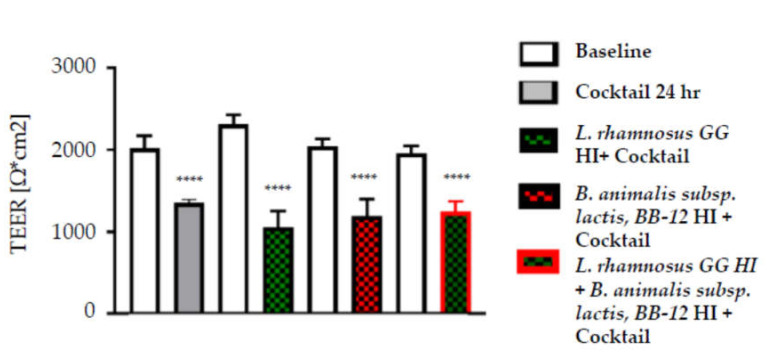
TEER measurements of Caco-2 cell cultures exposed to a cocktail of inflammatory cytokines following 24 h pretreatment with heat-inactivated (HI) *L. rhamnosus* GG or *B. animalis* subsp. *lactis*, BB-12 or their combination. *L. rhamnosus* GG was heat-inactivated for 3 min at 70 °C while or *B. animalis* subsp. *lactis,* BB-12 was heat-inactivated for 6 min at 62.3 °C. Data are mean + SEM (*n* = 9). **** *p* < 0.0001 vs. baseline.

Incubation with the inflammatory cocktail reduced TEER by 30% at 24 h and by 50% at 48 h (both *p* < 0.001) (Figure 3). Pre-incubation for 24 h with heat-inactivated *L. rhamnosus* GG or heat-inactivated *B. animalis* subsp. *lactis,* BB-12 or their combination did not alter the effect of the inflammatory cocktail on TEER (Figure 4).

### 3.5. Effect of Pre-Exposure to Heat-Inactivated L. rhamnosus GG and B. animalis subsp. Lactis, BB-12 on the Production of Inflammatory Mediators by Caco-2 Cells in Response to an Inflammatory Cocktail

**Figure 5:**
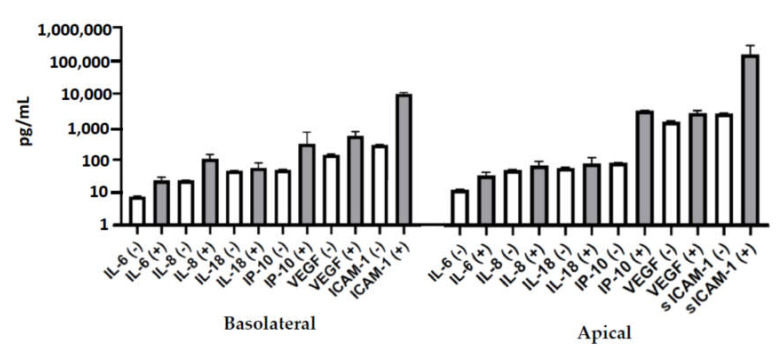
Cytokine concentrations (pg/mL) in the basolateral and the apical medium of Caco-2 cells without (white bars “(−)”) or with (grey bars “(+)”) 24 h treatment with an inflammatory cocktail on the basolateral side.

The inflammatory cocktail significantly increased production of all inflammatory mediators measured (Figure 5). IL-6, IL-18, and IP-10 concentrations were similar on both apical and basolateral sides. IL-8, VEGF, and sICAM-1 concentrations were higher on the apical than the basolateral side. IL-8 concentration was higher on the basolateral than the apical side.

**Figure 6:**
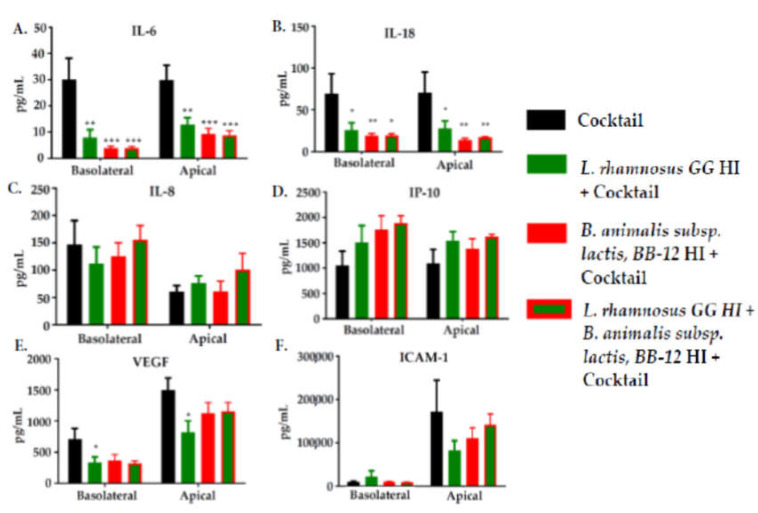
Concentrations (pg/mL) of IL-6 (**A**), IL-18 (**B**), IL-8 (**C**), IP-10 (**D**), VEGF (**E**), and sICAM-1 (**F**) in the basolateral and the apical medium of Caco-2 cells incubated on the apical side with heat-inactivated (HI) *L. rhamnosus GG*, *B. animalis* subsp. *lactis,* BB-12 or their combination for 24 h prior to 24 h treatment with an inflammatory cocktail on the basolateral side. *L. rhamnosus* GG was heat-inactivated for 3 min at 70 °C, while *B. animalis* subsp. *lactis,* BB-12 was heat-inactivated for 6 min at 62.3 °C. Data are mean + SEM (n = 9). One-way ANOVA *p* value 0.0003 (IL-6 both basolateral and apical), 0.012 (IL-18 basolateral), 0.008 (IL-18 apical), 0.05 (VEGF basolateral), 0.07 (VEGF apical), > 0.15 (all others). * *p* < 0.05, ** *p* < 0.01, *** *p* < 0.001 vs. cocktail alone.

IL-6 and IL-18 concentrations were decreased significantly in both the basolateral and the apical medium by pre-treatment of Caco-2 cells with heat-inactivated *L. rhamnosus* GG, heat-inactivated *B. animalis* subsp. *lactis,* BB-12 or their combination prior to stimulation with the inflammatory cocktail (Figure 6). Heat-inactivated *L. rhamnosus* GG and heat-inactivated *B. animalis* subsp. *lactis,* BB-12 had similar effects. There were also some significant effects of heat-inactivated *L. rhamnosus* GG and the combination of heat-inactivated *L. rhamnosus* GG and heat-inactivated *B. animalis* subsp. *lactis,* BB-12 on VEGF concentration on the apical and the basolateral sides, respectively. IL-8, IP-10, and sICAM-1 concentrations were not affected by heat-inactivated *L. rhamnosus* GG, heat-inactivated *B. animalis* subsp. *lactis*, BB-12 or the combination.

## 4. Discussion

Both probiotics used here (*L. rhamnosus* GG and *B. animalis* subsp. *lactis*, BB-12) increased TEER values in Caco-2 cell monolayers after 22 h of culture, indicating a strengthening of the epithelial monolayer when compared with the control condition. The effect was much greater for live organisms than for heat-inactivated organisms. This suggests a direct benefit from the live organisms on the epithelium, one that heat-inactivated organisms cannot fully exert, and that for an optimal beneficial effect on the epithelium, live organisms are required. The difference in effect between live and heat-inactivated organisms suggests that there may be two separate mechanisms of interaction of these bacteria with the epithelium, one physical (and seen with heat-inactivated organisms) and one metabolic (and thus requiring live organisms). Despite the clear effect of *L. rhamnosus* GG and *B. animalis* subsp. *lactis*, BB-12 on TEER, neither organism, either live of heat-inactivated, affected production of six inflammatory markers by unstimulated cultured Caco-2 cells.

Probiotics produce a number of metabolic products such as bacteriocins, acetaldehydes, and short-chain fatty acids, which contribute to the maintenance of enterocyte integrity [19,20,21]. This allows probiotics to exert biological activity not only by inhibiting the pathogenic growth of microbes in the host (bacteriocins) but through the strengthening of tight junctions, as described by others [22,23] and as suggested by the enhancement of TEER seen in the current research. The biological basis for any effect of heat-killed organisms has been described as an activity exerted through components in their cell walls, such as lipoteichoic acids [24] and peptidoglycan [25]. The active interaction between the bacterial strains and the host mucosal immune system and enterocytes differs and is specific according to bacterial properties. The differences observed between *L. rhamnosus* GG and *B. animalis* subsp. *lactis*, BB-12, confirming the observations of others [26], are likely due to intrinsic properties of each organism, such as the presence of the pili in *L. rhamnosus* GG [27], the composition of the cell wall with components such as lipoteichoic acid [24], and the presence of proteins that contribute to coping with stress conditions such as heat-inactivation [28].

The effect of live *L. rhamnosus* GG on TEER was stronger than that of live *B. animalis* subsp. *lactis*, BB-12. One of the mechanisms by which *L. rhamnosus* GG seems to interact with epithelial cells in a more effective manner than *B. animalis* subsp. *lactis*, BB-12 is through its pili structure, mainly because the pili allow closer and stronger interaction with the enterocytes, while *B. animalis* subsp. *lactis*, BB-12 lack this structure [5]. The findings suggest that the pili structure in the viable microorganism is partially responsible for a better interaction with the enterocytes. Although it has been suggested that the heat-inactivation does not destroy this structure [28], it might potentially reduce its ability to function. Another mechanism of action by which *L. rhamnosus* GG is acknowledged to enhance barrier function is through major secreted proteins p40 and p75 shown to protect against epithelial barrier disruption in vitro and ex vivo [29,30]. Live organisms are also known to release trophic factors that interact with enterocytes (e.g., lactic acid and short chain fatty acids), which may play a significant part in maintaining barrier integrity. For *L. rhamnosus* GG, the results of the current study suggest that those metabolic products may be more important for the interaction with epithelial cells than the physical interaction with components of the bacterial cell wall. For *B. animalis* subsp. *lactis*, BB-12, on the other hand, the TEER only dropped from an AUC of 207 to 142 when exposing Caco-2 cells with viable compared to completely heat-inactivated bacteria. This indicates that, for *B. animalis* subsp. *lactis*, BB-12, the cell wall components also present in the heat-inactivated bacteria may play an important role when interacting with epithelial cells.

Although it has been claimed that these strains in their inactivated form are safer than the active form in immunocompromised individuals [31,32], precisely due to their lack of metabolic activity and lack of potential overgrowth, the findings from the TEER measurements indicate that heat-inactivated microorganisms have reduced interaction with epithelial cells, at least from the epithelial barrier integrity standpoint. The lack of effect of *L. rhamnosus* GG and *B. animalis* subsp. *lactis*, BB-12 on inflammatory mediator production, even when alive, suggests that the nature of the interaction that increases TEER does not enhance or suppress the inflammatory response of gut epithelial cells. In vivo, the gut barrier includes a significant immune cell component that might respond differently from the epithelial cells [33]. Future in vitro experiments should explore these other interactions. Furthermore, adding immune cells such as dendritic cells into a co-culture system with the Caco-2 cells can allow a better picture of inflammatory/immune responses to probiotic bacteria, as these may require such immune-epithelial cross-talk [34].

This research did not explore bacterial modifications following heat-inactivation or bacterial components present in the co-culture media. Others have reported the effect of heat treatment of *L. rhamnosus* on its structure and physical features [35,36], although those authors used more severe conditions than in the current study. Nevertheless, it has been suggested that the adhesion of inactivated *B. animalis subsp. lactis,* BB-12 is considerable, even upon inactivation at 80 °C [37]. Moreover, a “probiotic paradox” theory proposes that both live and dead cells are able to induce advantageous biological responses [38]. Sugahara et al. compared immune-modulating properties of live and heat-inactivated *B. breve* in a mouse model and showed that suppression in production of pro-inflammatory cytokines and altered gene expression were seen for both bacterial forms [39].

The current study did not test mechanisms associated with direct contact of probiotic organisms with the epithelial cells or metabolites produced in cultures or whether the enhancement of TEER relates to an increase in junctional proteins. Despite the clear effect of *L. rhamnosus* GG and *B. animalis* subsp. *lactis*, BB-12 on TEER, neither organism, either live or heat-inactivated, affected production of six inflammatory mediators by unstimulated cultured Caco-2 cells. This suggests a divergence in the signaling mechanisms that lead to tight junction integrity and to production of inflammatory mediators, and that *L. rhamnosus* GG and *B. animalis* subsp. *lactis*, BB-12 can affect the former but not the latter.

Having established effects of *L. rhamnosus* GG and *B. animalis* subsp. *lactis*, BB-12 on TEER but not on inflammatory mediator production, the ability of the two organisms, when heat-inactivated, to prevent the effects of an inflammatory stimulus (a cocktail of three cytokines) on these outcomes was investigated. In preliminary experiments, we tested the effect of the individual components of the cytokine cocktail and of various combinations of these components using cytokine production as the readout; these experiments showed that the cocktail of three cytokines used here gave greater responses than the individual components or combination of two components (data not shown). The inflammatory cocktail itself decreased TEER, suggesting a breakdown in epithelial integrity, and increased production of inflammatory mediators, especially IL-6, IL-8, IP-10, and ICAM-1. In these experiments, the Caco-2 cells were exposed to the heat-inactivated probiotics for 24 h and then exposed to the inflammatory cocktail for a further 24 h. Pre-treatment with the heat-inactivated organisms did not prevent the effects of the inflammatory cocktail on TEER. However, inflammatory cocktail induced production of IL-6 and IL-18, in particular, was decreased. Once again, these observations suggest a divergence in the signaling mechanisms that lead to tight junction integrity and to the production of inflammatory mediators. In these experiments, the effects of heat-inactivated *L. rhamnosus* GG and *B. animalis* subsp. *lactis*, BB-12 were similar. The findings suggest that heat-inactivated *L. rhamnosus* GG or *B. animalis* subsp. *lactis*, BB-12, or their combination, could be used to protect the intestine from an inflammatory insult. We did not test whether the organisms could promote recovery from a pre-existing inflammatory state, but that would be very interesting in the context of treatment of a range of gastrointestinal conditions with probiotics.

Other researchers have suggested that heat-inactivated *L. rhamnosus* GG enhances epithelial barrier integrity through increased expression of the tight junction protein zonula occludens and therefore increase junctional complexes [40]. The mentioned research was conducted in an animal model of colitis, where an examination of the mucus layer was also performed. It is plausible that the mechanism behind this benefit was the stimulation of mucus-producing cells by heat-inactivated *L. rhamnosus* GG, so that the increased integrity was observed via increased mucus production. Further experiments using the Caco-2 cell model could be performed by adding mucus-producing cells to examine their interaction. It has been shown that a reduction in pro-inflammatory cytokine production by Caco-2 cells is a contributory factor in the protection of the epithelial barrier [41]. The current study identified a reduction in cytokine production but no effect on epithelial integrity as assessed by TEER and thus indicates that these two responses may not always be linked.

The results of the current study showed that the heat-inactivated organisms caused a reduction in the appearance of IL-6 on both basolateral and apical sides of the Caco-2 cells when added before the cytokine cocktail. TNF-α and IL-1β, components of the stimulating cytokine cocktail, activate the transcription factors that induce IL-6 gene expression [42]. The observed reduction in IL-6 production suggests that the Caco-2 cell response to the heat-inactivated bacteria interferes with that signaling pathway. IL-6 is a pleiotropic cytokine with several effects on the immune response in the gut [43], as IL-6 receptors are expressed in intestinal cells [44]. This cytokine has been found in gut biopsies of patients with inflammatory disease and coeliac disease as well as in healthy controls. In these biopsies, the protein was predominantly found in enterocytes [45]. These observations implicate IL-6 in intestinal damage and disease. However, other investigations have shown that IL-6 is also implicated in tissue repair, as some therapies blocking the effects of IL-6 cause damage to the intestine [46]. Thus, although the effects of the heat-inactivated organisms on IL-6 production may be viewed as anti-inflammatory and therefore of benefit, a clear conclusion of their health or clinical impact cannot be made.

IL-18 was also decreased by heat-inactivated *L. rhamnosus* GG and *B. animalis* subsp. *lactis*, BB-12 on both the basolateral and apical sides when the inflammatory cocktail was added. Inflammasomes are a complex of proteins that emerge during infections or tissue damage; they are induced by inflammatory cytokines [47]. Inflammasomes rapidly activate the release of IL-18 [47,48]. The observations made here suggest that the Caco-2 cell response to the heat-inactivated bacteria interferes with the signaling pathway that leads to inflammasome activation or activity. Generally, IL-18 is relevant in the signaling within intestinal epithelial cells that activates further inflammatory responses [49].

VEGF production was decreased on the basolateral side by both heat-inactivated organisms and on the apical side by heat-inactivated *L. rhamnosus* GG. Release of VEGF by epithelial cells indicates a relationship with, and regulation of, endothelial cells [22]. VEGF has shown to be increased in the intestinal mucosa of patients with active inflammatory bowel disease, Crohn’s disease, and ulcerative colitis [50]. Thus, a reduction in VEGF production by enterocytes could be clinically relevant.

The current study used the Caco2 cell line, perhaps the most widely studied model of gut epithelial cells. However, it is important to recognize that these cells are epithelial colorectal adenocarcinoma cells. Nevertheless, they do possess many features of native gut epithelial cells; they spontaneously differentiate to a polarized columnar epithelium and express the functional characteristics of mature intestinal enterocytes. Polarized Caco-2 cell monolayers show TEER values that are more similar to the in vivo situation than seen with some other gut epithelial cell lines. Caco-2 cells also express most receptors, transporters, and drug metabolizing enzymes found in normal gut epithelium. However, there are some limitations to the Caco-2 cell model. Firstly, as mentioned above, the normal epithelium contains more than one cell type (i.e., not only enterocytes), although this would be a limitation of any epithelial cell grown in isolation. Secondly, when using the Caco-2 cell model, no mucus or unstirred water layer is present.

## 5. Conclusions

*L. rhamnosus* GG and *B. animalis* subsp. *lactis,* BB-12 act on Caco-2 cells to increase TEER, an indicator of epithelial integrity, but they do not directly affect inflammatory mediator production of otherwise unstimulated cultures. Heat-inactivation of *L. rhamnosus* GG and *B. animalis* subsp. *lactis,* BB-12 markedly decreases their ability to increase TEER. Heat-inactivated *L. rhamnosus* GG and *B. animalis* subsp. *lactis,* BB-12 partially prevent the inflammatory cytokine-induced production of IL-6 and IL-18, and to a lesser extent VEGF, by Caco-2 cells but do not prevent the inflammation-induced decline in TEER. Heat-inactivated *L. rhamnosus* GG and *B. animalis* subsp. *lactis,* BB-12 can therefore have biological actions most likely due to structural features that are preserved after heat-inactivation. Inflammatory signaling to interrupt epithelial integrity and to elicit inflammatory cytokine production follow divergent pathways, and only the latter is sensitive to heat-inactivated *L. rhamnosus* GG and *B. animalis* subsp. *lactis,* BB-12. These findings are practically relevant because they indicate that live and, to a lesser extent, heat-inactivated *L. rhamnosus* GG and *B. animalis* subsp. *lactis,* BB-12 may strengthen the gut epithelial barrier and that the heat-inactivated organisms may prevent adverse inflammatory responses of the gut epithelium. Thus, heat-inactivated *L. rhamnosus* GG and *B. animalis* subsp. *lactis,* BB-12 may have food and nutraceutical applications.

## Figures and Tables

**Table 1:** Effect of heat-treatment for different times on numbers of *L. rhamnosus* GG and *B. animalis* subsp. *lactis*, BB-12.

Strain	Heat-Treatment at 62.3 °C	CFU/mL
*B. animalis* subsp. *lactis*, BB-12	0 min	9.4 × 10^8^
*B. animalis* subsp. *lactis*, BB-12	2 min	3.9 × 10^8^
*B. animalis* subsp. *lactis*, BB-12	4 min	1.2 × 10^5^
*B. animalis* subsp. *lactis*, BB-12	6 min	0
*B. animalis* subsp. *lactis*, BB-12	8 min	0
*L. rhamnosus* GG	0 min	7.5 × 10^8^
*L. rhamnosus* GG	2 min	6.8 × 10^8^
*L. rhamnosus* GG	4 min	3.9 × 10^8^
*L. rhamnosus* GG	6 min	1.1 × 10^8^
*L. rhamnosus* GG	8 min	2.2 × 10^7^
**Strain**	**Heat-Treatment at 70 °C**	**CFU/mL**
*L. rhamnosus* GG	0 min	8.9 × 10^8^
*L. rhamnosus* GG	1 min	5.5 × 10^8^
*L. rhamnosus* GG	3 min	0
*L. rhamnosus* GG	5 min	0
